# Within‐ and Between‐Channel Gaps Elicit Mismatch Negativity in the Aging Brain

**DOI:** 10.1111/ejn.70575

**Published:** 2026-06-09

**Authors:** Victoria Duda, Sayeed Devraj‐Kizuk, Erica Ogier

**Affiliations:** ^1^ School of Speech Pathology and Audiology Université de Montréal Montreal Canada; ^2^ Centre de Recherche Interdisciplinaire en Réadaptation (CRIR) Montréal Canada

**Keywords:** event‐related potentials, gap detection, mismatch negativity, temporal processing

## Abstract

This study examined how older and younger adults process silent gaps in auditory stimuli by recording cortical evoked potentials using a multi‐deviant paradigm that is compared to a psychophysical gap detection task. Participants passively listened to pairs of noise markers separated by silent intervals. Markers were either spectrally identical (within‐channel) or spectrally distinct (between‐channel) narrowband noises. Seven gap durations served as deviants in a multi‐deviant sequence. The deviance‐related negativity (DRN) and the P2/P3a were recorded from fronto‐central electrodes. Thirty‐two participants with normal hearing or minimal hearing loss participated in this study. They were separated into an older adult (mean age = 63 years) and younger adult (mean age = 24 years) group. Gapped deviants elicited DRN in both within‐ and between‐channel conditions. Age effects emerged in the DRN and peak‐to‐peak DRN‐P2/P3a measure. Older adults showed longer DRN latencies and reduced amplitudes compared to the younger group. Condition effects showed contrasting DRN latency patterns between groups. P2/P3a responses alone did not show any condition or age‐specific effects. Behavioral gap detection thresholds did not differ across conditions in older adults. Overall, results demonstrate that electrophysiological indices reveal subtle neural alterations in temporal resolution that may precede behavioral decline.

## Graphical abstract

This study examined the cortical responses of older and younger adults with normal hearing to silent gaps using EEG and behavioral gap detection. Behavioral gap detection thresholds did not differ across conditions in older adults. DRN and peak‐to‐peak DRN‐P2/P3a demonstrate subtle neural alterations in temporal resolution that may precede behavioral decline.

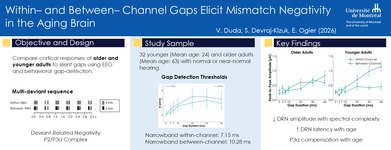

**Abbreviations**
DRN: deviance‐related negativity
MMN: mismatch negativity
APD: auditory processing disorder
GDT: gap detection threshold
RGDT: random gap detection test
GIN: gaps‐in‐noise
ERPs: event‐related potentials
OA: older adult
YA: younger adult
ER: Etymotics Research
WC‐NBN: within‐channel narrowband noise
bc‐NBN: between‐channel narrowband noise
EOG: electro‐oculogram
EEG: electroencephalography
ROI: region of interest
ANOVA: analysis of variance
CRUNCH: Compensation‐Related Utilization of Neural Circuits Hypothesis

## 1 Introduction

Successful verbal communication depends on the integrity of central auditory processing mechanisms that decode the temporal and spectral properties of speech. Temporal resolution, defined as the ability to detect brief acoustic changes such as silent gaps within an ongoing stimulus, is a fundamental aspect of auditory processing (Plomp 1964; Penner 1977). Clinically, temporal resolution is commonly assessed using behavioral measures such as the random gap detection test (RGDT; Keith 2000) and the gaps‐in‐noise (GIN) test (Musiek et al. 2005; Shinn et al. 2009). These paradigms typically use within‐channel stimuli, such as pure tones or broadband noise, in which the acoustic properties remain constant before and after the gap. However, natural speech contains dynamic spectral transitions and discontinuities between acoustically distinct segments. Such conditions are better approximated by between‐channel stimuli, in which the spectral characteristics differ across the silent interval. Studies consistently show elevated gap detection thresholds for between‐channel compared to within‐channel conditions, suggesting greater neural and perceptual processing demands when spectral continuity is disrupted (Heinrich et al. 2004; Lister et al. 2007; Lister et al. 2011).

Aging is associated with declines in temporal auditory processing, even in the absence of substantial peripheral hearing loss. Older adults typically demonstrate elevated behavioral gap detection thresholds relative to younger adults, with deficits becoming particularly pronounced in between‐channel conditions (Bertoli et al. 2002; Roberts and Lister 2004; Palmer and Musiek 2014). Pichora‐Fuller et al. (2006) further reported that age‐related declines are greater for spectrally asymmetrical markers, suggesting that aging disproportionately affects between‐channel temporal processing. These deficits may contribute to the speech perception difficulties frequently experienced by older listeners in complex acoustic environments.

Interpretation of behavioral gap detection performance is complicated by the contribution of non‐sensory cognitive factors, including attention, working memory, and motivation. Some evidence suggests that older adults recruit additional attentional resources during auditory tasks to compensate for degraded sensory input (Bertoli et al. 2002; Alain et al. 2004), whereas other studies indicate that age‐related declines in sustained attention may adversely affect task performance (Staub et al. 2013; Zanto and Gazzaley 2014). Electrophysiological methods provide a more objective means of evaluating auditory temporal processing because they do not require an overt behavioral response. In particular, the mismatch negativity (MMN), an event‐related potential (ERP) generated primarily in auditory cortical regions, reflects the automatic detection of acoustic change and is elicited largely independent of attention (Näätänen et al. 1978; Muller‐Gass et al. 2006; Sussman 2007).

The MMN is commonly elicited using an oddball paradigm in which infrequent deviant stimuli are interspersed among repetitive standard stimuli. Deviants differing in frequency, intensity, duration, or location reliably evoke the MMN, which typically peaks between 100 and 250 ms following stimulus onset. Silent gaps embedded within ongoing stimuli can also elicit MMN responses and have therefore been used as objective indices of temporal resolution (Bertoli et al. 2002; Todd et al. 2011). However, gap deviants additionally evoke an obligatory N1 response associated with the acoustic onset following the gap. Because the N1 temporally overlaps with the MMN, the resulting waveform is often referred to as a deviant‐related negativity (DRN) (Wei et al. 2002; Kimura and Takeda 2013). The present study adopts this terminology.

To improve recording efficiency, multi‐deviant paradigms have been developed in which several deviant types are presented within a single sequence (Näätänen et al. 2004). Duda‐Milloy et al. (2019) adapted this approach for gap detection by presenting six gap durations ranging from 2 to 40 ms within the same paradigm. DRN amplitudes obtained using this multi‐deviant design were comparable to those recorded using traditional oddball paradigms, supporting the validity and efficiency of the approach. Larger or more salient deviants may additionally elicit a later fronto‐central positivity, commonly identified as the P3a, which has been associated with involuntary attentional orienting toward unexpected acoustic events (Escera et al. 1998; Wetzel et al. 2013). In gap paradigms, however, this positivity may overlap with the P2 component, resulting in a composite P2/P3a response (Duda et al. 2025; Augereau et al. 2025).

Several studies have reported reduced MMN amplitudes and prolonged latencies in older adults, suggesting age‐related declines in automatic auditory discrimination (Gaeta et al. 1998; Bertoli et al. 2002; Getzmann and Näätänen 2015). In a gap‐specific MMN study, Bertoli et al. (2002) demonstrated that older adults required longer gaps to elicit cortical responses and exhibited smaller MMN amplitudes and delayed latencies relative to younger adults. Importantly, however, this work focused exclusively on within‐channel stimuli. To our knowledge, no study has directly compared cortical responses to within‐ and between‐channel gap stimuli across younger and older adults using MMN‐based paradigms.

Current theoretical accounts of MMN are strongly influenced by predictive coding frameworks, which propose that the auditory system continuously generates predictions regarding incoming sensory input and updates these internal models when prediction errors occur (Friston 2005; Garrido et al. 2009). Within this framework, gap detection reflects not only the encoding of an acoustic interruption but also the integrity of predictive and integrative auditory processes. Between‐channel gaps likely impose greater computational demands because they require integration of spectrally discontinuous information across time and frequency channels (Heinrich et al. 2004; Lister et al. 2011). Aging may weaken these predictive and integrative mechanisms, resulting in noisier sensory representations, delayed neural responses, and reduced sensitivity to rapid acoustic change (Bertoli et al. 2002; Getzmann and Näätänen 2015). Nevertheless, the interaction between aging and spectral complexity in cortical gap processing remains poorly understood.

The present study used a multi‐deviant gap paradigm to examine how gap duration, spectral complexity, and age influence cortical auditory responses. Specifically, we compared DRN and P2/P3a responses elicited by within‐channel and between‐channel gap stimuli in younger and older adults with normal hearing. We hypothesized that between‐channel conditions would elicit smaller DRN and P2/P3a amplitudes than within‐channel conditions because of increased perceptual and neural processing demands. We further hypothesized that older adults would exhibit reduced amplitudes relative to younger adults, consistent with prior evidence of age‐related declines in cortical temporal processing (Bertoli et al. 2002).

## 2 Methods

### 2.1 Participants

A total of 32 participants volunteered to participate in this study. The participants were separated into an older adult (OA) and a younger adult (YA) group. The ages for the OA group ranged from 51 to 77 years (mean = 63 years, SD = 6.8), while the ages for the YA group ranged from 19 to 31 years (mean = 24 years, SD = 3.0). Hearing levels were verified using standard audiological testing. All participants had hearing thresholds below 40 dB HL from 250 to 8000 Hz. None reported a history of tinnitus, dizziness, neurological disorder, psychiatric disorder, or uncontrolled chronic health disorder. Procedures were approved by the University of Montreal Clinical Research Ethics Committee in accordance with Law 21 of the Civil Code of Québec, according to the Tri‐Council Policy Statement guide and the Good Clinical Practices and the Policy on Research with Human Beings (60.1). Based on these guidelines, all participants were explained the nature of this study, gave written consent, and received an honorarium for their participation.

### 2.2 Procedure and Stimuli

The auditory stimuli were constructed using Audacity software, Version 3.0.2. The auditory stimuli were presented through a Solid State Logic SSL‐2 audio interface. E‐Prime (Psychology Software Tools, Version 3.0) software was used for the timing and presentation of the auditory stimuli. They were presented binaurally through ER2A insert headphones while the participant sat in a sound and electrically isolated booth. The response curve of the ER2A earphones is flat from 20 to 16 kHz. The participants were asked to watch a silent, subtitled film of their choice and thus to ignore the auditory stimuli.

**FIGURE 1:**
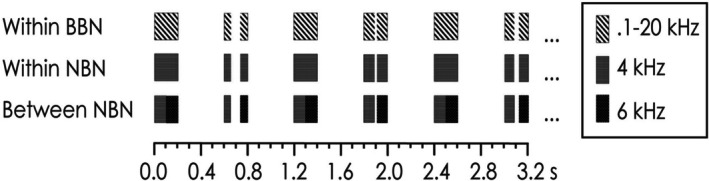
**Multi‐deviant auditory sequences under three stimulus conditions.** Auditory sequences were presented using two types of noise stimuli: within‐channel and between‐channel noise. In each condition, the standard stimulus was either a passband‐filtered noise centered at 4 kHz or a combination of passband‐filtered noise split between 6 and 4 kHz. Deviant stimuli matched their respective standards, except for a brief silent gap inserted at the temporal midpoint. Standard and deviant sounds alternated throughout each sequence, with seven gap durations introduced in pseudorandom order. *Note:* Gap durations are not drawn to scale.

The multi‐deviant sequence consisted of the presentation of a standard alternating with deviants, similar to that used by Duda‐Milloy et al. (2019) (see Figure 1). All standards were a 200‐ms filtered Gaussian white noise, presented at 80 dB SPL and with an instantaneous rise‐and‐fall time. Two different conditions were run. In a within‐channel condition (WC‐NBN), the standard was a 4‐kHz narrowband noise. In a between‐channel condition (bc‐NBN), the standard was a continuous 6‐kHz narrowband noise for the first 100 ms and 4‐kHz narrowband noise for the last 100 ms. High‐frequency stimuli were selected because these frequencies support the perception of speech sounds, particularly fricatives and other high‐frequency consonants, that are critical for speech clarity (Shadle et al. 2023). Seven deviant stimuli were created by inserting a silent interval (a gap) in the center of the standard (i.e., around 100 ms). The duration of the gap was either 2, 5, 7, 10, 20, 30, or 40 ms, each having an instantaneous onset and offset. In younger adults, this permitted the inclusion of the examination of sub‐threshold (2 ms), near‐threshold (5–7 ms), and supra‐threshold (20–40 ms) gaps (Duda‐Milloy et al. 2019). The inter‐stimulus interval (offset‐to‐onset) was 400 ms.

Because the standard and the deviant stimuli alternated, each was presented with a probability of 0.5. The probability of occurrence of any one of the seven deviants was 0.083. The first 10 presentations in each sequence were standard stimuli in order to strengthen its memory trace. The standard stimulus was presented 549 times (including the initial 10 standards), and the deviants were thus presented 539 times per sequence (77 presentations of each deviant). The order of occurrence of deviants was pseudorandomized, such that in an array of seven deviants, the same deviant was not presented consecutively and the order of deviants within the array was never the same throughout the sequence. The timing and presentation of the stimuli were controlled by a computer running E‐Prime (Psychology Software Tools, Version 3.0) software. The sequences were presented two times for each condition. The order of sequences was counterbalanced across participants. A brief break was provided between sequences.

Following the presentation of the multi‐deviant sequences, participants from the OA group were asked to actively attend to the auditory stimuli and to detect the occurrence of a stimulus containing a gap. In this behavioral gap detection paradigm, a total of 40 standards and 20 of each of the seven deviants were presented for each condition. The order of the standards and deviants was randomized. Participants were asked to push one mouse button if they did not hear a gap and another mouse button if they did hear a gap in the stimulus. When the participants failed to respond to either the standard or the deviant, the data for that trial were rejected. This occurred on an average of 34.5% of trials for the standards and 7.3% of trials for the deviants.

### 2.3 EEG/ERP Recording

The electroencephalography (EEG) was recorded from 32 active Ag/AgCl electrodes attached to an ActiCap electrode cap (Brain Products GmbH, Munich, Germany) using a standard 10–10 layout. Vertical eye movements and blink artifacts were recorded from an electrode placed on the infra‐orbital ridge of the right eye. An average reference was used for all channels, including the electro‐oculogram (EOG). Inter‐electrode impedances were kept between 20 and 60 kΩ. The amplifier hardware low pass filter was set at 249 Hz, and the high pass filter was set at 0.08 Hz. The electrode signals were digitized continuously at a 500‐Hz sampling rate and stored on disk for later analyses.

The data were analyzed using Brain Products BrainVision Analyser 2.3 and plotted using Python v3.13. A vertical EOG channel was computed by subtracting activity recorded at FP2 from that of the EOG located on the infra‐orbital ridge, and a horizontal EOG channel was computed by subtracting FT9 activity from that of FT10. The EEG and EOG data were digitally filtered using a zero‐phase‐shift Butterworth IIR filter with a low cutoff of 1 Hz and a high cutoff of 20 Hz. An additional notch filter at 60 Hz was also used to attenuate sources of electrical noise. Continuous EEG data were epoched into 800 ms single‐trial segments, beginning 200 ms before stimulus onset. After epoching, regression‐based ocular correction (Gratton et al. 1983) was used to remove the influence of eye movements from the EEG channels. Baseline correction was then performed, using the average of all activity in the 100 ms before stimulus onset was used as the pre‐stimulus baseline. Epochs that exceeded a maximal voltage step of 50 μV/ms or in which the maximum voltage difference exceeded 200 μV were rejected from further analyses. Finally, epochs were averaged for each participant and event type and exported for further analysis.

### 2.4 Statistical Analysis

The onset of both the standard and deviant stimuli elicited the obligatory N1 and P2 responses. The deviant also elicited additional ERP responses, notably the DRN and, in some cases, the P2/P3a. These deviant‐specific deflections are best observed in a difference wave, computed by subtracting the point‐by‐point average ERP elicited by the standard from that elicited by the deviant. This subtraction procedure removes responses common to both stimulus types, thereby isolating responses unique to the deviant features.

For individual participants, the DRN was quantified as the mean amplitude within a ± 25 ms window centered on the peak latency identified in their individual average waveform. To determine whether each deviant stimulus elicited a statistically reliable DRN, confidence intervals were calculated around the group mean of the difference wave at each deviant duration. A deviant was considered to elicit a significant negativity if the upper limit of the 95% confidence interval fell below 0 μV. This approach is statistically equivalent to performing a one‐sample *t*‐test comparing the mean difference waveform to zero (Winer et al. 1971). The analyses were run at a fronto‐central region of interest (ROI: Fz, Cz, FC1, and FC2 sites) where the DRN typically reaches its maximum amplitude. For the individual effects of gap duration and condition on DRN amplitude, a three‐way ANOVA was conducted with one between factor, group (younger and older adults), and two within factors, type of deviant (seven durations) and condition (WC‐NBN and bc‐NBN). When a significant main effect of gap duration was observed, post hoc comparisons were conducted using a Bonferroni correction. For the group effects of age and condition on the DRN amplitude, a three‐way mixed analysis of variance (ANOVA) was used with condition and gap duration as the within factors and age as a between factor.

Behavioral performance was quantified using the signal detection measure *d*′, which is computed on the basis of both hit (a deviant containing a gap correctly detected) and false alarm (a standard not containing a gap falsely detected as containing a gap) rates (Macmillan and Creelman 1991). A one‐way repeated‐measures ANOVA was performed on *d*′ scores across the seven gap durations, with Bonferroni corrections used for post hoc comparisons. Finally, Spearman correlation coefficients were calculated to assess the relationship between DRN amplitude and behavioral sensitivity (*d*′) at each gap duration.

## 3 Results

### 3.1 Neurophysiological Data

#### 3.1.1 Standard ERP

**FIGURE 2:**
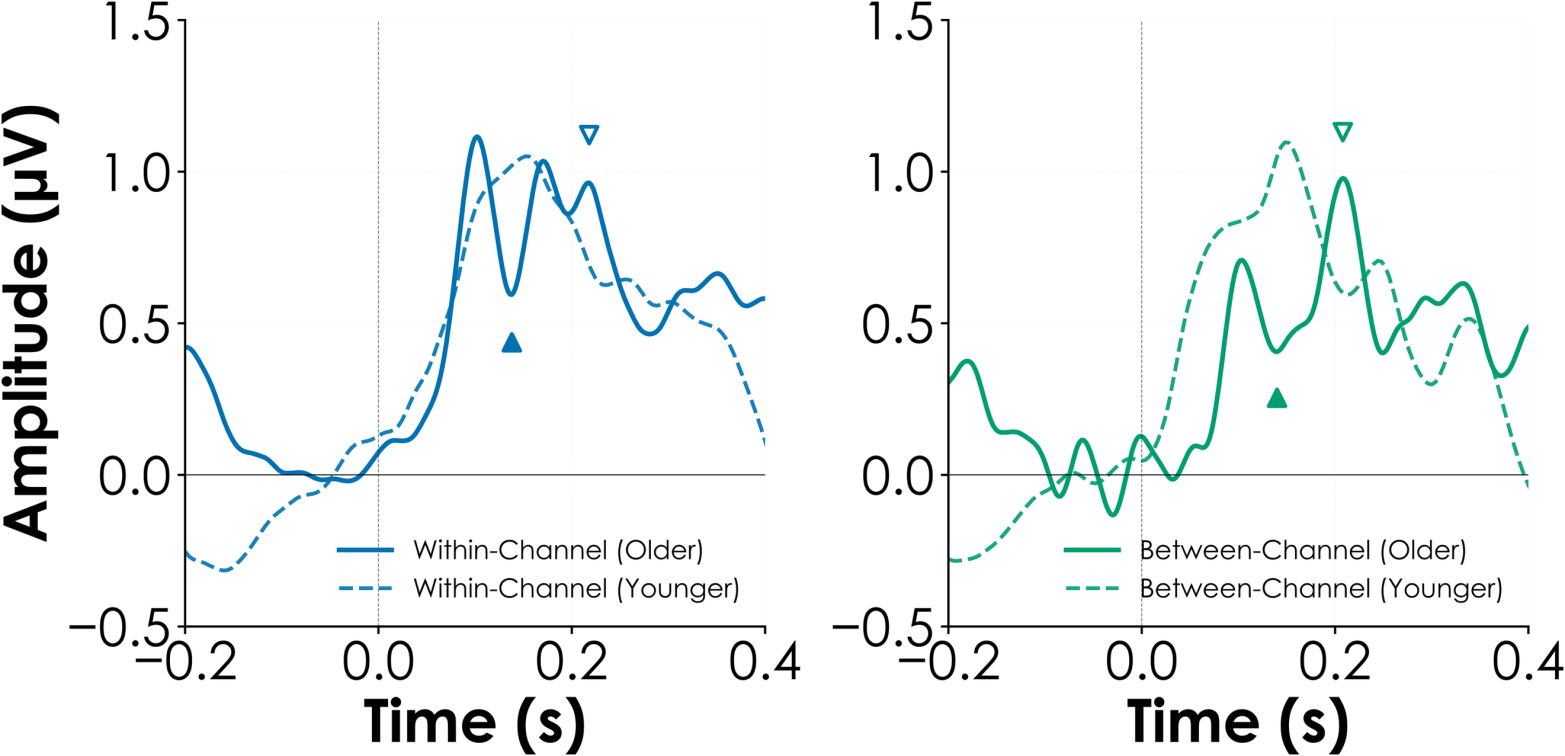
**Standard ERP waveforms and component identification across stimulus conditions.** Grand‐averaged event‐related potential (ERP) responses of young (dashed blue) and older adults (dashed green) are shown for two conditions: within‐channel narrowband (blue) and between‐channel narrowband (green). Recordings were taken from the fronto‐central region of interest (ROI), including electrodes Fz, Cz, FC1, and FC2. Two key components were detected: the N1 (138–144 ms), indicated by a filled triangle, and the P2 (206–220 ms), indicated by an unfilled triangle.

The DRN was measured in a standard‐deviant difference wave. The use of the difference wave assumes that the DRN was elicited by the deviant stimulus and that differences in the DRN between the WC‐NBN and the bc‐NBN were a result of differential processing of the deviant. This assumption is valid only if processing of the standard did not vary between the conditions. To ensure that any differences observed in the DRN can be attributed to differential processing of the deviants alone, a comparison of the standard stimuli across conditions was first conducted. The ERPs evoked by the standard stimulus for each of the conditions are presented in Figure 2. In this figure, a negative‐going deflection is observed at about 138 to 140 ms (OA) and 120 ms (YA), followed by a positive‐going deflection at about 208–218 ms (OA) and 180 ms (YA). These are the N1 and P2 deflections that were elicited by the onset of the standard. A repeated‐measures ANOVA was performed to determine group differences and the effect of condition.

The effect of condition at ROI (Fz, Cz, FC1, and FC2) was not significant for the N1 amplitude (*p* = 0.157). There was no significant group difference (*p* = 0.083). The condition × age interaction was also not significant (*p* = 0.128). For the standard P2, there was no significant effect of condition (*p* = 0.328), group (*p* = 0.605), nor condition × age interaction (*p* = 0.743). These results are expected given that the first 100 ms of the deviant was physically identical to the standard. The effect of the conditions is thus the same for the standard and the deviant for the initial processing of the stimuli.

#### 3.1.2 Difference Waves Across Condition

**FIGURE 3:**
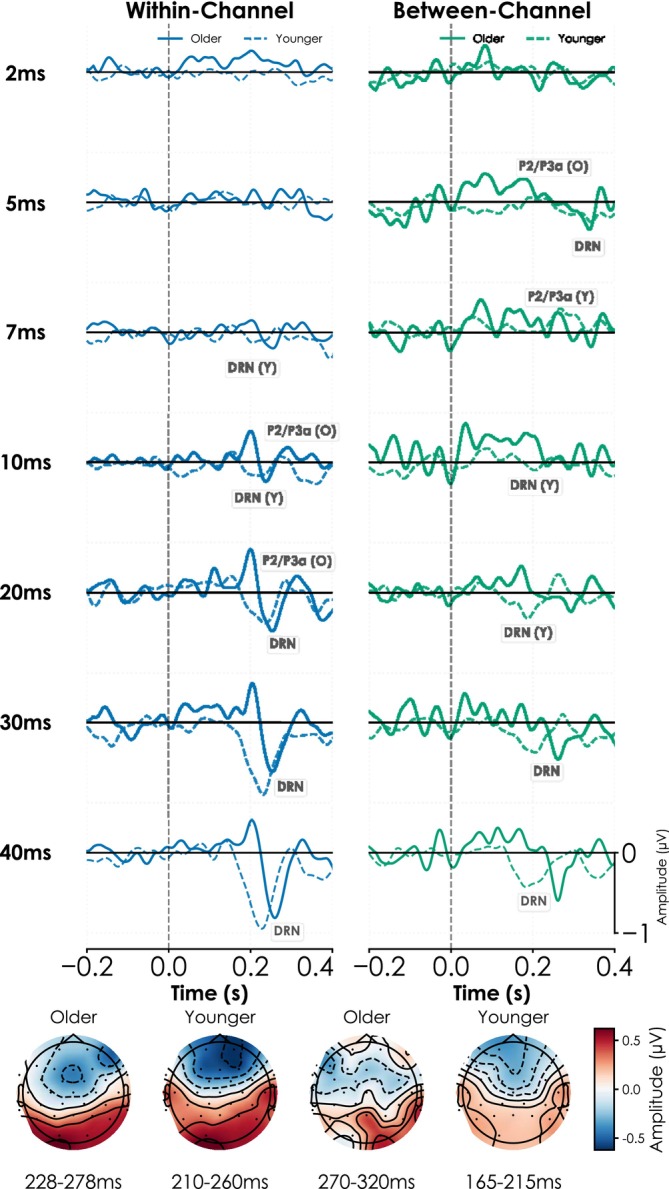
**DRN difference waveforms across stimulus conditions and gap durations.** Upper panel: Each subplot presents grand‐averaged difference waveforms (deviant minus standard) for gap stimuli of the young adults (YA: dashed line) and older adults (OA: solid line) under two conditions: narrowband within‐channel (blue) and narrowband between‐channel (green). Recordings were obtained from the fronto‐central region of interest (ROI), comprising electrodes Fz, Cz, FC1, and FC2. The deviance‐related negativity (DRN), observed between 240–290 ms (OA) and 185–240 ms (YA), is indicated by an upward arrow. P2/P3a components (OA: 290–350 ms, YA: 256–300 ms) are marked with a downward arrow. Note that only significant DRN or P2/P3a are indicated. Larger waveform amplitudes reflect stronger mismatch detection. Robust DRN responses are evident across both conditions, with amplitude increasing proportionally to gap duration. Lower panel: Scalp topographies for the 40 ms gap duration in WC‐NBN and bc‐NBN conditions, shown with DRN latency. A fronto‐central negativity accompanied by temporal positivity is observed. Responses are generally stronger in the WC‐NBN condition within each group, with the negativity appearing more centralized in the older group than in the younger group.

Figure 3 shows the grand average difference waveforms elicited by the seven deviants in the WC‐NBN and the bc‐NBN conditions. A distinct negativity, the DRN, was elicited from 240 to 290 ms (OA) and from 185 to 240 ms (YA) after the onset of the stimulus. The DRN was maximum in amplitude over the fronto‐central areas of the scalp (see lower panel of Figure 3). It inverted in amplitude at the mastoids. The amplitude of the DRN was significantly more negative in younger than in older adults overall (older mean = −0.24 μV, younger mean = −0.34 μV; *t* = 2.25, *p* = 0.025). Confidence interval testing (at the ROI) revealed that significant DRNs were obtained for more gap durations in younger than in older adults. In older adults, the DRN was significantly negative for the 20, 30, and 40 ms gaps in the WC‐NBN condition and for the 5, 30, and 40 ms gaps in the bc‐NBN condition. In younger adults, the DRN reached significance for the 7, 10, 20, 30, and 40 ms gaps in the WC‐NBN condition and for the 5, 10, 20, 30, and 40 ms gaps in the bc‐NBN condition.

A subsequent positivity was also evident with longer duration gaps. This positivity peaked between 290–350 ms (OA) and 256–300 ms (YA) after the onset of the longer duration gaps. It is probably an obligatory P2, but a P3a has also been known to be elicited for the longer duration gaps (Tavakoli et al. 2021). It will therefore be labeled as a composite P2/P3a. Confidence interval testing indicated that the P2/P3a amplitude was significantly different from baseline for the 10 and 20 ms gaps in the WC‐NBN condition for older adults. The P2/P3a was not significantly different from baseline at any deviant in the WC‐NBN condition for younger adults. In the bc‐NBN condition, the deviants reaching significance were the 5 ms gap for older adults and the 7 ms gap for younger adults.

Because the assumption of sphericity was violated, Greenhouse–Geisser corrections were applied, when possible. As expected, a main effect of gap duration was observed (*F*_4.19,125.79_ = 22.02, MSE = 0.22, *p* < 0.001, η^2^_p_ = 0.42). The amplitude of the DRN generally increased as the duration of the gap increased. Bonferroni‐corrected post hoc comparisons revealed that the 2 ms gap elicited significantly smaller DRN amplitudes than the 30 and 40 ms gaps; the 5 ms gap differed from the 30 and 40 ms gaps; the 7 ms gap differed from the 20, 30, and 40 ms gaps; the 10 ms gap differed from the 30 and 40 ms gaps; and the 20 ms gap differed from the 40 ms gap (all *p* ≤ 0.05). A significant main effect of condition was also observed (*F*_1,30_ = 8.85, MSE = 0.26, *p* = 0.006, η^2^_p_ = 0.23). The DRN was significantly reduced in the bc‐NBN condition. The gap duration × condition interaction was also significant (*F*_3.91, 117.44_ = 5.37, MSE = 0.22, *p* < 0.001, η^2^_p_ = 0.15), reflecting larger gap‐duration effects under the WC‐NBN condition than under bc‐NBN. The group main effect was significant (*F*_1,30_ = 4.33, MSE = 0.26, *p* = 0.046, η^2^_p_ = 0.13), with younger adults showing larger DRN amplitudes overall than older adults. The group × gap interaction was not significant (*p* = 0.56) nor was the condition × group interaction (*p* = 0.19). The gap × condition × group three‐way interaction was also not significant (*p* = 0.29).

DRN latencies were analyzed across age groups, conditions, and gap durations, revealing several significant effects. A main effect of group was observed (*F*_1,30_ = 611.16, MSE = 350.34, *p* < 0.001, η^2^_p_ = 0.95) with older participants exhibiting significantly later DRNs than younger participants. A main effect of gap duration was also significant (*F*_4.80, 144.12_ = 59.99, MSE = 292.40, *p* < 0.001, η^2^_p_ = 0.67), with smaller gaps eliciting later DRNs. The main effect of condition was not significant (*F*_1, 30_ = 0.26, MSE = 234.51, *p* = 0.612). All two‐ and three‐way interactions reached significance: the condition × group interaction (*F*_1, 30_ = 28.62, MSE = 234.51, *p* < 0.001, η^2^_p_ = 0.49), the gap × group interaction (*F*_4.80, 144.12_ = 21.31, MSE = 292.40, *p* < 0.001, η^2^_p_ = 0.42), the condition × gap interaction (*F*_4.81, 144.34_ = 45.51, MSE = 280.63, *p* < 0.001, η^2^_p_ = 0.60), and the condition × gap × group three‐way interaction (*F*_4.81, 144.34_ = 81.97, MSE = 280.63, *p* < 0.001, η^2^_p_ = 0.73). To clarify the nature of these interactions, post hoc comparisons were conducted. Within‐channel and between‐channel DRN latencies differed in both age groups but in opposite directions: In older participants, the bc‐NBN DRN peaked later than the WC‐NBN DRN by 8.5 ms (*p* < 0.001), whereas in younger participants the WC‐NBN DRN peaked later than the bc‐NBN DRN by 7.0 ms (*p* = 0.002). Across all gap durations, older participants consistently showed longer DRN latencies than younger participants.

**FIGURE 4:**
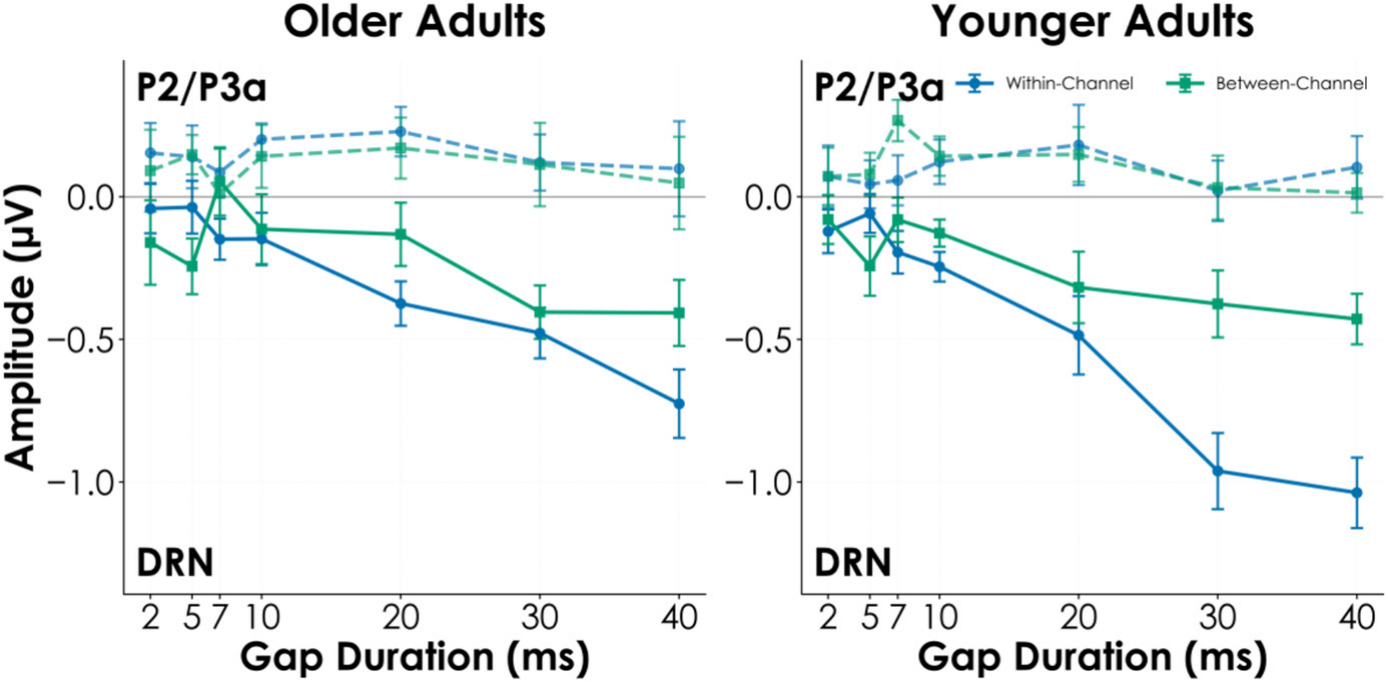
**Mean deviant amplitudes at fronto‐central ROI.** Grand‐averaged amplitudes of the DRN (solid lines) and P2/P3a (dashed lines) components as a function of gap duration (2–40 ms) in older (left) and younger adults (right). Results are shown separately for the WC‐narrowband (blue) and bc‐narrowband (green) conditions. Error bars represent ±1 standard error of the mean (SEM). Both age groups show a general decrease in DRN amplitude with increasing gap duration, with a steeper decline under the WC‐narrowband condition, particularly in younger adults.

P2/P3a amplitudes were also compared with the gap duration (see Figure 4). This positivity did not show a significant main effect of gap (*F*_4.7,142.43_ = 0.85, MSE = 0.17, *p* = 0.515). The main effect of condition was also not significant (*F*_1,30_ = 0.05, MSE = 0.37, *p* = 0.83). The main effect of group was not significant (*F*_1,30_ = 0.2, MSE = 0.40, *p* = 0.69), and there were no significant interactions: age × condition (*F*_1,30_ = 0.35, MSE = 0.37, *p* = 0.56), gaps × group (*F*_4.75,142.43_ = 0.61, MSE = 0.17, *p* = 0.68), conditions × gap (*F*_3.09,92.58_ = 0.17, MSE = 0.34, *p* = 0.92), and condition × group × gap (*F*_3.09,92.58_ = 0.29, MSE = 0.34, *p* = 0.839).

#### 3.1.3 Peak‐To‐Peak Amplitude as a Function of Gap Duration and Stimulus Condition

**FIGURE 5:**
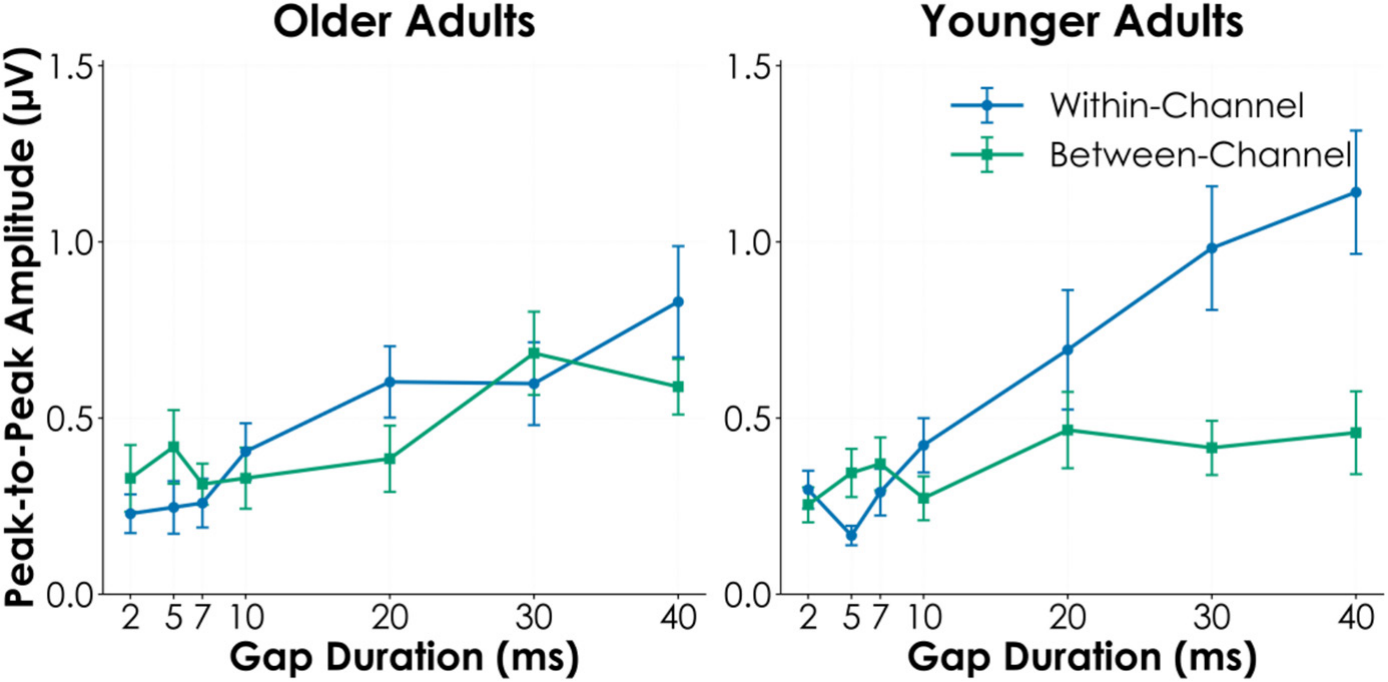
**Peak‐to‐peak amplitudes (DRN‐P2/P3a) as a function of gap duration (2–40 ms) in older (left) and younger adults (right).** Results are shown separately for the WC‐NBN (blue) and bc‐NBN (green) conditions. Vertical bars represent the standard error (SE) around the mean. In both age groups, amplitudes generally increased with longer gap durations, but the effect was stronger in the WC‐NBN condition, particularly in younger adults. In contrast, amplitudes in the bc‐NBN condition remained relatively stable across gap durations.

As is apparent in Figure 2, the pre‐stimulus zero voltage baseline was not stable. An alternative peak‐to‐peak scoring method was used instead of a baseline‐to‐peak measure as done in previous papers (Duda et al. 2020, 2025). The peak of the DRN was measured with respect to the subsequent positive peak. The mean peak‐to‐peak data are presented in Figure 5.

This showed a significant main effect of gap duration (*F*_3.23, 96.83_ = 20.80, MSE = 0.22, *p* < 0.001, η^2^_p_ = 0.41). The main effect of condition was also significant (*F*_1, 30_ = 5.52, MSE = 0.24, *p* = 0.03, η^2^_p_ = 0.16). The gap duration × condition interaction was significant (*F*_4.56, 136.89_ = 7.91, MSE = 0.13, *p* < 0.001, η^2^_p_ = 0.21). The analysis revealed a significant Age × Condition × Gap Duration interaction for peak‐to‐peak amplitudes (*F*_4.56, 136.89_ = 2.85, MSE = 0.13, *p* = 0.021, η^2^_p_ = 0.09). Simple‐effects analyses on the three‐way interaction revealed that, in younger adults under the WC‐NBN condition, peak‐to‐peak amplitudes increased substantially across gap durations: The smaller gaps (2–10 ms) differed from the largest gaps (30 and 40 ms; all *p* ≤ 0.005). In older adults under the WC‐NBN condition, only the largest gaps (30 and 40 ms) consistently differed from the smaller ones (2–10 ms vs. 40 ms, all *p* ≤ 0.04). In the bc‐NBN condition for both age groups, no gap‐pair contrasts survived Bonferroni correction. In Figure 5, in older adults, amplitudes increased with increasing gap duration in both conditions, but the increase was more pronounced in the WC‐narrowband condition. In younger adults, the WC‐NBN condition shows a significantly steeper increase in amplitude with longer gaps, whereas the bc‐NBN condition remains relatively stable across gap durations.

### 3.2 Behavioral Data

**FIGURE 6:**
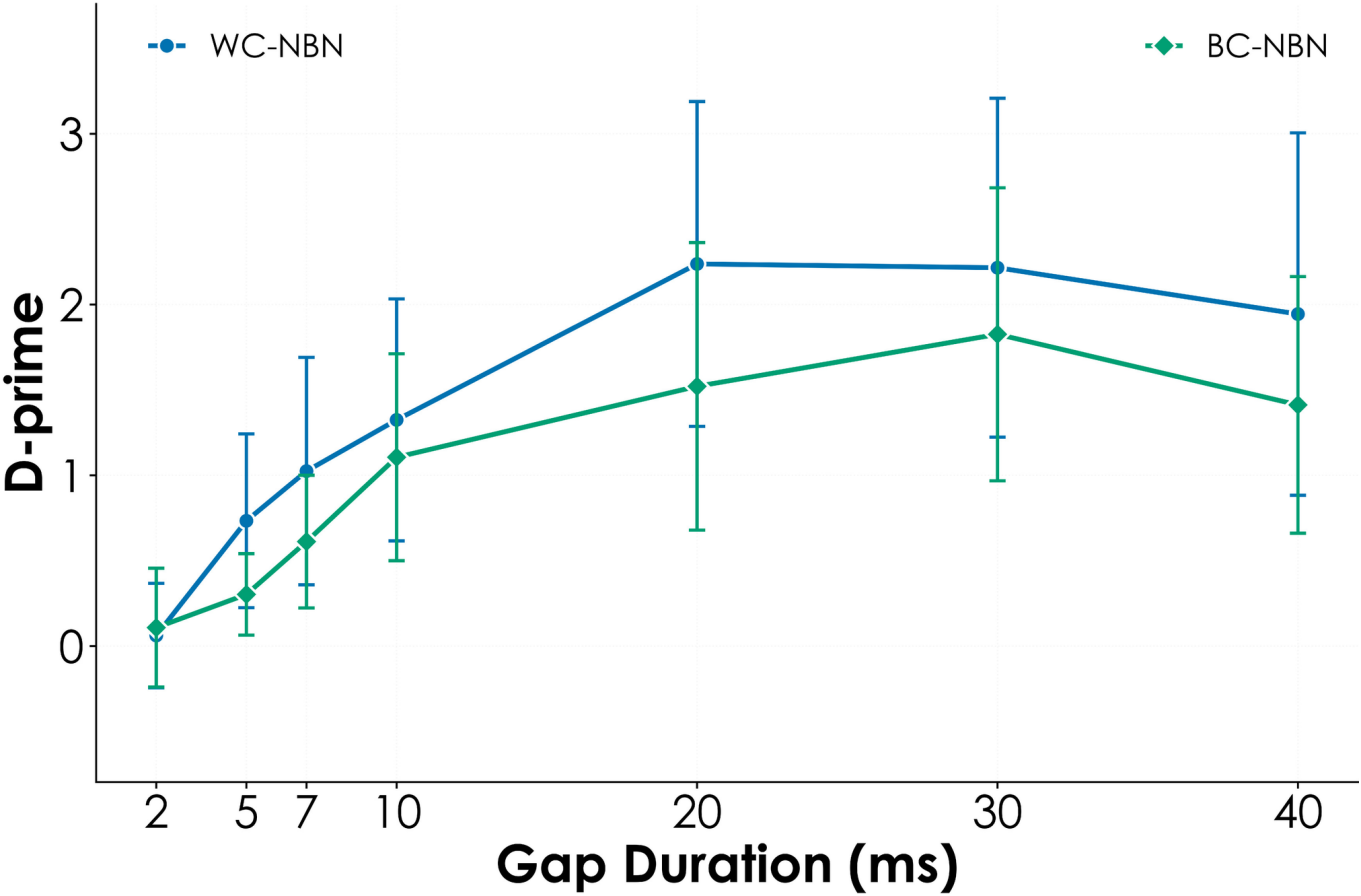
**Gap detection accuracy across stimulus conditions in older adults.** Mean sensitivity index (*d*‐prime) values are plotted against gap duration for three types of stimuli: within‐channel narrowband noise (blue) and between‐channel narrowband noise (green). Each line represents one stimulus condition, with data points showing participant averages and error bars indicating plus or minus the standard error of the mean. All conditions show improved sensitivity with longer gaps.

Figure 6 illustrates how the *d*′ scores vary as a function of gap duration and condition in only the older adults. Seven participants were removed from the analysis because of incomplete data. The main effect of gap duration was significant (*F*_6, 48_ = 11.48, MSE = 0.97, *p* < 0.001, η^2^_p_ = 0.59). The *d*′ index increased systematically with gap duration; however, after Bonferroni correction, no specific gap‐duration pair reached the *p* < 0.05 threshold, reflecting limited power at *n* = 9. The main effect of condition was not significant (*F*_1, 8_ = 1.33, MSE = 4.53, *p* = 0.28, η^2^_p_ = 0.14). On average, *d*′ was numerically higher in the WC‐NBN condition (*M* = 1.36) than in the bc‐NBN condition (*M* = 0.98), but this difference did not reach significance. The condition × deviant interaction was also not significant (*F*_6, 48_ = 1.40, MSE = 0.26, *p* = 0.23, η^2^_p_ = 0.15).

The average gap detection threshold (GDT), computed per participant by linear interpolation at *d*′ = 1, was 7.15 ms (SD = 3.93, *n* = 7) for the WC‐NBN condition and 10.28 ms (SD = 7.09, *n* = 6) for the bc‐NBN condition. Two to three older participants did not reach *d*′ = 1 within the tested gap range (2–40 ms) and were excluded from the threshold mean for those conditions. A paired‐samples *t*‐test in the six participants with thresholds in both conditions revealed no significant difference between the two GDTs, *t*(5) = −0.65, *p* = 0.545.

#### 3.2.1 Correlations Between *d*′ and DRN

A Spearman correlation was calculated on the *d*′ measures of perceptual sensitivity and the peak‐to‐peak gap ERP amplitude using a one‐tailed test of significance. It was expected that an increase in amplitude would be related to an increase in behavioral perceptibility as noted by Duda‐Milloy et al. (2019) in young adult populations. Pooling across the seven deviants (*n* = 63 observations per condition; 9 participants × 7 gap durations), the Spearman correlation was significant for the WC‐NBN condition (rho = 0.34, *p* = 0.003, one‐tailed) and not significant for the bc‐NBN condition (rho = 0.07, *p* = 0.30, one‐tailed).

## 4 Discussion

The objective of this study was to investigate electrophysiological and behavioral responses to gap duration and to evaluate how stimulus complexity influences the DRN and P2/P3a components in younger and older adults with normal hearing. Behaviorally, older adults showed no significant gap detection threshold differences between within‐ and between‐channel narrowband conditions, whereas electrophysiological results revealed clear condition‐ and/or age‐dependent variations in the DRN latency and amplitude and peak‐to‐peak DRN‐P2/P3a amplitude. The behavioral *d*′ was correlated with the within‐channel narrowband condition.

### 4.1 Within‐ and Between‐Channel Gap Changes of MMN/DRN

This study demonstrates that gapped deviants generally modulate DRN amplitude and latency at frontal electrodes in both within‐ and between‐channel narrowband noise conditions. The DRN was robust for gaps above 10 ms, increased in amplitude with longer gaps, and was reduced in amplitude in the between‐channel condition. These findings are consistent with MMN and DRN studies showing gap‐related cortical responses that scale with perceptual salience (Näätänen et al. 2007; Duda‐Milloy et al. 2019; Duda et al. 2025; Augereau et al. 2025; Takegata et al. 2009).

These findings also align with predictive‐coding accounts of auditory temporal processing, which suggest that gap detection reflects the interaction between bottom‐up encoding and top‐down temporal predictions (Winkler and Schröger 2015). The reduced DRN amplitude and prolonged latency observed in the between‐channel condition suggest that integrating discontinuous spectral information imposes greater computational demands on the auditory system, consistent with models emphasizing the role of higher order auditory cortex in cross‐channel integration (Heinrich et al. 2004; Lister et al. 2011).

### 4.2 Age‐Related Changes of MMN/DRN

Our data contribute to the literature by revealing age‐ and condition‐specific differences. Older adults showed generally longer DRN latencies and smaller amplitudes across both WC‐NBN and bc‐NBN compared to younger adults. Similar observations have been made in Bertoli et al. (2002) who also found reduced MMN amplitudes and increased latencies in elderly subjects compared to younger participants using within‐channel gapped stimuli. Augereau et al. (2025) also found that in middle‐aged adults DRN latencies were prolonged for several gap durations. Together, these results showing age‐related MMN changes to gapped stimuli support the hypothesis that aging weakens predictive and integrative mechanisms, resulting in slower model updating and less efficient encoding of rapid acoustic change (Getzmann and Näätänen 2015; Bertoli et al. 2002; Augereau et al. 2025).

The opposite latency patterns observed across age groups suggest that younger and older adults rely on fundamentally different neural strategies when processing within‐ and between‐channel gaps. In older adults, the longer DRN latency for between‐channel compared to within‐channel gaps likely reflects the greater computational demands associated with integrating spectrally discontinuous cues, a process known to rely on higher order auditory regions and to be particularly vulnerable to age‐related declines in temporal precision and predictive updating (Pekkonen et al. 1993, 1996). Similar findings have been reported in Lister et al. (2011) who showed prolonged latencies of the N1 in a between‐channel gap condition compared to a within‐channel condition in older participants. In contrast, our results show younger adults presented a reverse pattern, with longer latencies for within‐channel gaps. In Heinrich et al. (2004), despite the absence of reported MMN latencies, the published group‐averaged waveforms for younger adults display a marked decrease in MMN latency for between‐channel gaps relative to within‐channel gaps. This may suggest that cross‐channel integration is relatively efficient in early adulthood, whereas detecting subtle temporal discontinuities within a single spectral channel may require more fine‐grained analysis. From a predictive‐coding perspective, these findings imply that aging slows the updating of internal auditory predictions when stimuli require integration across spectral channels, while younger listeners generate larger and later prediction‐error responses for within‐channel deviations. The consistent latency prolongation in older adults across all gap durations further supports the view that early cortical temporal processing slows with age and that the added spectral complexity of between‐channel stimuli imposes an additional processing cost on an already taxed system.

### 4.3 P2/P3a Age Effects for Within‐ and Between‐Channel Gaps

The P2/P3a alone was not systematically modulated by gap duration, and no age‐related differences emerged when these components were analyzed independently. The P2/P3a effects in our study were subtle and condition‐specific in older adults, with amplitudes peaking around 10–20 ms in the within‐channel condition (unlike the younger group) and a peri‐threshold gap in the between‐channel condition. This diverges somewhat from Tavakoli et al. (2021), who found maximal responses for the longest gaps, but parallels Augereau et al. (2025), who reported robust P2/P3a responses in middle‐aged adults. Taken together, these findings suggest that orienting responses (indexed by P2/P3a) are preserved with age but are shaped by stimulus complexity and salience. Age‐related patterns became apparent when examining the peak‐to‐peak DRN‐P2/P3a measure, suggesting that age‐related differences may not always be visible at the level of single ERP components but may emerge in the temporal interplay of successive processes.

The presence of P2/P3a in older adults is noteworthy. P2 has been linked to early attentional processes, sensory gating, and stimulus evaluation (Crowley and Colrain 2004; Luck 2014). The P2/P3a in older adults during within‐channel conditions may reflect altered sensory filtering and less efficient inhibitory control, consistent with age‐related changes reported in Go/NoGo tasks and novelty paradigms (Vallesi et al. 2009; Pfefferbaum et al. 1980). This interpretation is reinforced by the emergence of the P3a in older but not younger adults in the within‐channel condition. The P3a, typically linked to involuntary orienting to novelty (Polich 2007; Escera et al. 1998), may reflect compensatory recruitment: For older adults, even relatively simple within‐channel gaps may pose higher perceptual demands, thereby engaging broader attentional networks. For younger adults, the same gaps may be resolved pre‐attentively without requiring additional resources, consistent with more efficient temporal resolution (Alain and McDonald 2007).

These results align with the CRUNCH model (Compensation‐Related Utilization of Neural Circuits Hypothesis; Reuter‐Lorenz and Cappell 2008), which posits that older adults compensate for declining efficiency by recruiting additional neural resources at lower task demands. In our study, the within‐channel gap condition, processed effortlessly by younger adults, elicited P3a activity in older adults, an indication of over‐recruitment. Importantly, this compensatory activity allowed for slightly larger DRN amplitudes and for behavioral performance to be preserved, as reflected in similar gap detection thresholds across conditions. However, the reduced condition sensitivity of older adults' DRN responses, coupled with fewer and delayed DRNs, suggests less precise neural encoding and a potential ceiling on compensation when task demands increase (Cabeza et al. 2018).

From a predictive‐processing perspective, these findings suggest that older adults may rely more heavily on compensatory attentional mechanisms because their internal auditory models are less precise and require more frequent updating. The emergence of P3a responses in older adults, even for relatively short gaps (10 and 20 ms), may reflect an increased need to recruit higher order networks to resolve prediction errors that younger adults can resolve pre‐attentively. This interpretation integrates the CRUNCH framework with models of auditory prediction, offering a unified account of why aging affects early sensory encoding and later attentional processes in distinct but interacting ways.

### 4.4 Relating Behavioral to Electrophysiological Data

Our behavioral findings generally replicate prior studies. Gap detection thresholds fell in the single‐digit millisecond range for within‐channel conditions (Heinrich et al. 2004; Moore et al. 1993), and thresholds were elevated in between‐channel conditions, as previously reported (Formby and Forrest 1991; Grose et al. 2001; Schneider and Hamstra 1999). However, the difference in perceptual sensitivity between conditions did not differ significantly. This dissociation between electrophysiological and behavioral measures deserves emphasis. While behavioral data did not differ significantly between conditions, neural responses showed clear condition‐ and age‐specific variations. This suggests that EEG indices may be more sensitive to early or subtle age‐related changes that are not yet reflected in psychophysics, consistent with prior work showing neural–behavioral decoupling in aging (Bidelman et al. 2014; Alain et al. 2018; Bertoli et al. 2002). There was, however, a significant correlation between the ERP amplitudes and the behavioral measures for the within‐channel condition but not for the between‐channel condition in older adults. This suggests that for complex tasks (such as the between‐channel task), neural inefficiencies may be masked by compensatory strategies that maintain performance, obscuring direct brain–behavior links.

The dissociation between behavioral and electrophysiological measures underscores the value of the multi‐deviant MMN/DRN paradigm for isolating sensory‐driven versus integrative components of temporal resolution. Whereas behavioral thresholds reflect the final outcome of perceptual decision‐making, potentially buffered by compensatory strategies, DRN and P2/P3a responses reveal the underlying neural computations. The selective sensitivity of DRN to spectral configuration suggests that this component indexes early sensory‐integrative processes, while the P2/P3a reflects downstream orienting and evaluative mechanisms (Näätänen et al. 2007; Crowley and Colrain 2004; Polich 2007). This layered pattern provides a more nuanced account of temporal resolution than behavioral measures alone can offer.

### 4.5 Study Limitations

Several limitations must be acknowledged. The modest sample size may have limited our ability to detect subtle effects, and our narrowband stimuli restrict generalization to real‐world contexts such as speech. Our participants all had near‐clinically normal hearing, so present results may underestimate deficits in older adults with hearing loss. Our older participant group was also younger than those typically reported in aging studies. For example, Bertoli et al. (2002) included an older cohort with a mean age of 72 years, whereas the mean age in our study was 65 years. This may pose a challenge when comparing the findings with other research groups. Future research should use larger, more diverse samples including clinical populations; incorporate naturalistic stimuli to probe ecological validity; apply source localization or connectivity analyses to identify compensatory networks; and use longitudinal designs to track whether age‐related differences reflect stable compensatory strategies or progressive decline.

## 5 Conclusion

This study provides electrophysiological evidence that spectral complexity modulates cortical gap processing differently across the adult lifespan. While previous work has documented age‐related changes in within‐channel gap detection, no study has directly contrasted within‐ and between‐channel processing using the DRN. Our results demonstrate that spectral discontinuity selectively reduces DRN amplitude and alters latency patterns and that these effects interact with age. This provides empirical support for the theoretical claim that between‐channel gaps place greater demands on integrative auditory mechanisms, demands that appear disproportionately challenging for older adults.

## Author Contributions

**Victoria Duda** led the conceptualization and methodology design of the project, secured funding, supervised the research personnel and graduate student team, managed project administration, and authored the original manuscript draft. **Sayeed Devraj‐Kizuk** oversaw laboratory procedures and protocol compliance, maintained and programmed equipment, conducted data analysis and validation, curated and stored research data, developed visualizations, and provided substantive edits to the draft. **Erica Ogier** collected participant data as part of her clinical Master's internship and contributed foundational content from her research report to the original draft.

## Funding

Financial support for this research was provided by a Discovery Grant obtained by V.D. from the Natural Sciences and Engineering Research Council of Canada (NSERC) (RGPIN‐2022‐04390).

## Conflicts of Interest

The authors declare no conflicts of interest.

## Supporting information


**Data S1:** Annex.pdf. **Annex.: Grand‐average ERP waveforms of younger and older adults across stimulus conditions for various gap durations.** Grand‐average ERP waveforms for younger and older adults in within‐channel (blue) and between‐channel (green) gap conditions across seven gap durations (2–40 ms). Each panel shows the deviant (solid line) and standard (dashed line) responses at the fronto‐central region of interest (ROI), comprising electrodes Fz, Cz, FC1, and FC2. Amplitude is plotted in microvolts (μV) and time in seconds (s) with positivity plotted upwards.

## Data Availability

The data that support the findings for this study are available from the corresponding author upon reasonable request.

## References

[ejn70575-bib-0001] Alain, C. , Y. Du , L. J. Bernstein , T. Barten , and K. Banai . 2018. “Listening Under Difficult Conditions: An Activation Likelihood Estimation Meta‐Analysis.” Human Brain Mapping 35, no. 4: 1693–1709. 10.1002/hbm.24031. PMC6866419 29536592

[ejn70575-bib-0002] Alain, C. , and K. L. McDonald . 2007. “Age‐Related Differences in Neuromagnetic Brain Activity Underlying Concurrent Sound Perception.” Journal of Neuroscience 27, no. 6: 1308–1314. 10.1523/JNEUROSCI.5433-06.2007. 17287505 PMC6673581

[ejn70575-bib-0003] Alain, C. , K. L. McDonald , J. M. Ostroff , and B. Schneider . 2004. “Aging: A Switch From Automatic to Controlled Processing of Sounds?” Psychology and Aging 19, no. 1: 125–133. 10.1037/0882-7974.19.1.125. 15065936

[ejn70575-bib-0004] Augereau, T. M. , D. Paromov , A. B. Lacerda , V. Duda , and F. Champoux . 2025. “Electrophysiological Markers of Early Auditory Temporal Resolution Deterioration With Aging.” Hearing Research 464: 109325. 10.1016/j.heares.2025.109325. 40494162

[ejn70575-bib-0005] Bertoli, S. , J. Smurzynski , and R. Probst . 2002. “Temporal Resolution in Young and Elderly Subjects as Measured by Mismatch Negativity and a Psychoacoustic Gap Detection Task.” Clinical Neurophysiology 113, no. 3: 396–406. 10.1016/S1388-2457(02)00013-5. 11897540

[ejn70575-bib-0006] Bidelman, G. M. , J. W. Villafuerte , S. Moreno , and C. Alain . 2014. “Age‐Related Changes in the Subcortical–Cortical Encoding and Perception of Speech Cues.” Journal of Neuroscience 34, no. 6: 2164–2175. 10.1016/j.neurobiolaging.2014.05.006. 24908166

[ejn70575-bib-0007] Cabeza, R. , M. Albert , S. Belleville , et al. 2018. “Maintenance, Reserve and Compensation: The Cognitive Neuroscience of Healthy Ageing.” Nature Reviews Neuroscience 19, no. 11: 701–710. 10.1038/s41583-018-0068-2. PMC6472256 30305711

[ejn70575-bib-0008] Crowley, K. E. , and I. M. Colrain . 2004. “A Review of the Evidence for P2 Being an Independent Component Process: Age, Sleep and Modality.” Clinical Neurophysiology 115, no. 4: 732–744. 10.1016/j.clinph.2003.11.021. 15003751

[ejn70575-bib-0009] Duda, V. , T. M. Augereau , K. Campbell , and A. Koravand . 2025. “Does Masking Alter the Mismatch Negativity Response to Gaps?” Brain Research 1859: 149651. 10.1016/j.brainres.2025.149651. 40274176

[ejn70575-bib-0010] Duda, V. , K. Campbell , and A. Koravand . 2020. “Event‐Related Potentials Following Gaps in Noise: The Effects of the Intensity of Preceding Noise.” Brain Research 1748: 147078. 10.1016/j.brainres.2020.147078. 32861677

[ejn70575-bib-0011] Duda‐Milloy, V. , P. Tavakoli , K. Campbell , D. L. Benoit , and A. Koravand . 2019. “A Time‐Efficient Multi‐Deviant Paradigm to Determine the Effects of Gap Duration on the Mismatch Negativity.” Hearing Research 377: 34–43. 10.1016/j.heares.2019.03.004. 30901627

[ejn70575-bib-0012] Escera, C. , K. Alho , E. Schröger , and I. Winkler . 1998. “Involuntary Attention and Distractibility as Evaluated With Event‐Related Brain Potentials.” Audiology and Neuro‐Otology 3, no. 2–3: 151–166. 10.1159/000013877. 10859410

[ejn70575-bib-0013] Formby, C. , and T. G. Forrest . 1991. “Detection of Silent Temporal Gaps in Sinusoidal Markers.” Journal of the Acoustical Society of America 89, no. 2: 830–837. 10.1121/1.1894643. 2016432

[ejn70575-bib-0014] Friston, K. 2005. “A Theory of Cortical Responses.” Philosophical Transactions of the Royal Society of London. Series B, Biological Sciences 360, no. 1456: 815–836. 10.1098/rstb.2005.1622. 15937014 PMC1569488

[ejn70575-bib-0015] Gaeta, H. , D. Friedman , W. Ritter , and J. Cheng . 1998. “An Event‐Related Potential Study of Age‐Related Changes in Sensitivity to Stimulus Deviance.” Neurobiology of Aging 19, no. 5: 447–459. 10.1016/S0197-4580(98)00087-6. 9880047

[ejn70575-bib-0016] Garrido, M. I. , J. M. Kilner , K. E. Stephan , and K. J. Friston . 2009. “The Mismatch Negativity: A Review of Underlying Mechanisms.” Clinical Neurophysiology: Official Journal of the International Federation of Clinical Neurophysiology 120, no. 3: 453–463. 10.1016/j.clinph.2008.11.029. 19181570 PMC2671031

[ejn70575-bib-0017] Getzmann, S. , and R. Näätänen . 2015. “The Mismatch Negativity as a Measure of Auditory Stream Segregation in a Simulated “cocktail‐party” Scenario: Effect of Age.” Neurobiology of Aging 36, no. 11: 3029–3037. 10.1016/j.neurobiolaging.2015.07.017. 26254109

[ejn70575-bib-0018] Gratton, G. , M. G. Coles , and E. Donchin . 1983. “A New Method for Off‐Line Removal of Ocular Artifact.” Electroencephalography and Clinical Neurophysiology 55, no. 4: 468–484. 10.1016/0013-4694(83)90135-9. 6187540

[ejn70575-bib-0019] Grose, J. H. , J. W. Hall III , E. Buss , and D. Hatch . 2001. “Gap Detection for Similar and Dissimilar Gap Markers.” Journal of the Acoustical Society of America 109, no. 4: 1587–1595. 10.1121/1.1354983. 11325129

[ejn70575-bib-0020] Heinrich, A. , C. Alain , and B. A. Schneider . 2004. “Within‐ and Between‐Channel Gap Detection in the Human Auditory Cortex.” Neuroreport 15, no. 13: 2051–2056. 10.1097/00001756-200409150-00011. 15486480

[ejn70575-bib-0021] Keith, R. W. 2000. Random Gap Detection Test. Auditec.

[ejn70575-bib-0022] Kimura, M. , and Y. Takeda . 2013. “Task Difficulty Affects the Predictive Process Indexed by Visual Mismatch Negativity.” Frontiers in Human Neuroscience 7: 267. 10.3389/fnhum.2013.00267. 23781189 PMC3679470

[ejn70575-bib-0023] Lister, J. J. , N. D. Maxfield , and G. J. Pitt . 2007. “Cortical Evoked Response to Gaps in Noise: Within‐Channel and Across‐Channel Conditions.” Ear and Hearing 28, no. 6: 862–878. 10.1097/AUD.0b013e3181576cba. 17982372 PMC4792277

[ejn70575-bib-0024] Lister, J. J. , N. D. Maxfield , G. J. Pitt , and V. B. Gonzalez . 2011. “Auditory Evoked Response to Gaps in Noise: Older Adults.” International Journal of Audiology 50, no. 4: 211–225. 10.3109/14992027.2010.526967. 21385014 PMC4788511

[ejn70575-bib-0025] Luck, S. J. 2014. An Introduction to the Event‐Related Potential Technique. 2nd ed. MIT Press.

[ejn70575-bib-0026] Macmillan, N. A. , and C. D. Creelman . 1991. Detection Theory: A User's Guide. Cambridge University Press.

[ejn70575-bib-0027] Moore, B. C. , R. W. Peters , and B. R. Glasberg . 1993. “Detection of Temporal Gaps in Sinusoids: Effects of Frequency and Level.” Journal of the Acoustical Society of America 93, no. 3: 1563–1570. 10.1121/1.406815. 8473610

[ejn70575-bib-0028] Muller‐Gass, A. , R. M. Stelmack , and K. B. Campbell . 2006. “The Effect of Visual Task Difficulty and Attentional Direction on the Detection of Acoustic Change as Indexed by the Mismatch Negativity.” Brain Research 1078, no. 1: 112–130. 10.1016/j.brainres.2005.12.125. 16497283

[ejn70575-bib-0029] Musiek, F. E. , J. B. Shinn , R. Jirsa , D. E. Bamiou , J. A. Baran , and E. Zaida . 2005. “GIN (Gaps‐In‐Noise) Test Performance in Subjects With Confirmed Central Auditory Nervous System Involvement.” Ear and Hearing 26, no. 6: 608–618. 10.1097/01.aud.0000188069.80699.41. 16377996

[ejn70575-bib-0030] Näätänen, R. , A. W. Gaillard , and S. Mäntysalo . 1978. “Early Selective‐Attention Effect on Evoked Potential Reinterpreted.” Acta Psychologica 42, no. 4: 313–329. 10.1016/0001-6918(78)90006-9. 685709

[ejn70575-bib-0031] Näätänen, R. , P. Paavilainen , T. Rinne , and K. Alho . 2007. “The Mismatch Negativity (MMN) in Basic Research of Central Auditory Processing: A Review.” Clinical Neurophysiology 118, no. 12: 2544–2590. 10.1016/j.clinph.2007.04.026. 17931964

[ejn70575-bib-0032] Näätänen, R. , S. Pakarinen , T. Rinne , and R. Takegata . 2004. “The Mismatch Negativity (MMN): Towards the Optimal Paradigm.” Clinical Neurophysiology 115, no. 1: 140–144. 10.1016/j.clinph.2003.04.001. 14706481

[ejn70575-bib-0033] Palmer, S. B. , and F. E. Musiek . 2014. “Electrophysiological Gap Detection Thresholds: Effects of Age and Comparison With a Behavioral Measure.” Journal of the American Academy of Audiology 25, no. 10: 999–1007. 10.3766/jaaa.25.10.8. 25514452

[ejn70575-bib-0034] Pekkonen, E. , V. Jousmäki , J. Partanen , and J. Karhu . 1993. “Mismatch Negativity Area and Age‐Related Auditory Memory.” Electroencephalography and Clinical Neurophysiology 87, no. 5: 321–325. 10.1016/0013-4694(93)90185-X. 7693443

[ejn70575-bib-0035] Pekkonen, E. , T. Rinne , K. Reinikainen , T. Kujala , K. Alho , and R. Näätänen . 1996. “Aging Effects on Auditory Processing: An Event‐Related Potential Study.” Experimental Aging Research 22, no. 2: 171–184. 10.1080/03610739608254005. 8735151

[ejn70575-bib-0036] Penner, M. J. 1977. “Detection of Temporal Gaps in Noise as a Measure of the Decay of Auditory Sensation.” Journal of the Acoustical Society of America 61, no. 2: 552–557. 10.1121/1.381297. 845317

[ejn70575-bib-0037] Pfefferbaum, A. , J. M. Ford , W. T. Roth , and B. S. Kopell . 1980. “Age‐Related Changes in Auditory Event‐Related Potentials.” Electroencephalography and Clinical Neurophysiology 49, no. 3–4: 266–276. 10.1016/0013-4694(80)90221-7. 6158403

[ejn70575-bib-0038] Pichora‐Fuller, M. K. , B. A. Schneider , N. J. Benson , S. J. Hamstra , and E. Storzer . 2006. “Effect of Age on Detection of Gaps in Speech and Nonspeech Markers Varying in Duration and Spectral Symmetry.” Journal of the Acoustical Society of America 119, no. 2: 1143–1155. 10.1121/1.2149837. 16521775

[ejn70575-bib-0039] Plomp, R. 1964. “Rate of Decay of Auditory Sensation.” Journal of the Acoustical Society of America 36, no. 2: 277–282. 10.1121/1.1918946.

[ejn70575-bib-0040] Polich, J. 2007. “Updating P300: An Integrative Theory of P3a and P3b.” Clinical Neurophysiology 118, no. 10: 2128–2148. 10.1016/j.clinph.2007.04.019. 17573239 PMC2715154

[ejn70575-bib-0041] Reuter‐Lorenz, P. A. , and K. A. Cappell . 2008. “Neurocognitive Aging and the Compensation Hypothesis.” Current Directions in Psychological Science 17, no. 3: 177–182. 10.1111/j.1467-8721.2008.00570.x.

[ejn70575-bib-0042] Roberts, R. A. , and J. J. Lister . 2004. “Effects of Age and Hearing Loss on Gap Detection and the Precedence Effect.” Journal of Speech, Language, and Hearing Research 47, no. 5: 965–978. 10.1044/1092-4388(2004/071). 15603455

[ejn70575-bib-0043] Schneider, B. A. , and S. J. Hamstra . 1999. “Gap Detection Thresholds as a Function of Tonal Duration for Younger and Older Listeners.” Journal of the Acoustical Society of America 106, no. 1: 371–380. 10.1121/1.427062. 10420628

[ejn70575-bib-0044] Shadle, C. H. , W. R. Chen , L. L. Koenig , and J. L. Preston . 2023. “Refining and Extending Measures for Fricative Spectra, With Special Attention to the High‐Frequency Range.” Journal of the Acoustical Society of America 154, no. 3: 1932–1944. 10.1121/10.0021075. 37768114 PMC10540850

[ejn70575-bib-0045] Shinn, J. B. , G. D. Chermak , and F. E. Musiek . 2009. “GIN (Gaps‐In‐Noise) Performance in the Pediatric Population.” Journal of the American Academy of Audiology 20, no. 4: 229–238. 10.3766/jaaa.20.4.3. 19927695

[ejn70575-bib-0046] Staub, B. , N. Doignon‐Camus , O. Després , and A. Bonnefond . 2013. “Sustained Attention in the Elderly: What Do We Know and What Does It Tell Us About Cognitive Aging?” Ageing Research Reviews 12, no. 2: 459–468. 10.1016/j.arr.2012.12.001. 23261761

[ejn70575-bib-0047] Sussman, E. S. 2007. “A New View on the MMN and Attention Debate: The Role of Context in Processing Auditory Events.” Journal of Psychophysiology 21, no. 3–4: 164–175. 10.1027/0269-8803.21.34.164.

[ejn70575-bib-0048] Takegata, R. , R. Heikkilä , and R. Näätänen . 2009. “Neural Process Underlying Gap Detection for Spectrally Rich and Asymmetrical Markers.” Neuroreport 20, no. 12: 1120–1124. 10.1097/WNR.0b013e32832eb80e. 19590396

[ejn70575-bib-0049] Tavakoli, P. , V. Duda , A. Boafo , and K. Campbell . 2021. “The Effects of Sleep on Objective Measures of Gap Detection Using a Time‐Efficient Multi‐Deviant Paradigm.” Brain and Cognition 152: 105772. 10.1016/j.bandc.2021.105772. 34218026

[ejn70575-bib-0050] Todd, J. , B. Finch , E. Smith , T. W. Budd , and U. Schall . 2011. “Temporal Processing Ability is Related to Ear‐Asymmetry for Detecting Time Cues in Sound: A Mismatch Negativity (MMN) Study.” Neuropsychologia 49, no. 1: 69–82. 10.1016/j.neuropsychologia.2010.10.029. 21040739

[ejn70575-bib-0051] Vallesi, A. , D. T. Stuss , A. R. McIntosh , and T. W. Picton . 2009. “Age‐Related Differences in Processing Irrelevant Information: Evidence From Event‐Related Potentials.” Neuropsychologia 47, no. 2: 577–586. 10.1016/j.neuropsychologia.2008.10.018. 19022270

[ejn70575-bib-0052] Wei, J. H. , T. C. Chan , and Y. J. Luo . 2002. “A Modified Oddball Paradigm “cross‐modal delayed response” and the Research on Mismatch Negativity.” Brain Research Bulletin 57, no. 2: 221–230. 10.1016/S0361-9230(01)00742-0. 11849829

[ejn70575-bib-0053] Wetzel, N. , E. Schröger , and A. Widmann . 2013. “The Dissociation Between the P3a Event‐Related Potential and Behavioral Distraction.” Psychophysiology 50, no. 9: 920–930. 10.1111/psyp.12072. 23763292

[ejn70575-bib-0054] Winer, B. J. , D. R. Brown , and K. M. Michels . 1971. Statistical Principles in Experimental Design. 2nd ed. p. 596. McGraw‐Hill. https://web.archive.org/web/20170808020238id_/http://tocs.ulb.tu‐darmstadt.de/7475330.pdf.

[ejn70575-bib-0055] Winkler, I. , and E. Schröger . 2015. “Auditory Perceptual Objects as Generative Models: Setting the Stage for Communication by Sound.” Brain and Language 148: 1–22. 10.1016/j.bandl.2015.05.003. 26184883

[ejn70575-bib-0056] Zanto, T. P. , and A. Gazzaley . 2014. “Attention and Ageing.” In The Oxford Handbook of Attention, edited by A. C. Nobre and S. Kastner , 927–971. Oxford University Press. 10.1093/oxfordhb/9780199675111.013.020.

