# Supplementary figures and images for: Within‐ and Between‐Channel Gaps Elicit Mismatch Negativity in the Aging Brain
Source: Eur J Neurosci. 2026 Jun 9;63(11):e70575. doi: 10.1111/ejn.70575 (PMC13250239; doi:10.1111/ejn.70575)

# Older Adults

# Younger Adults

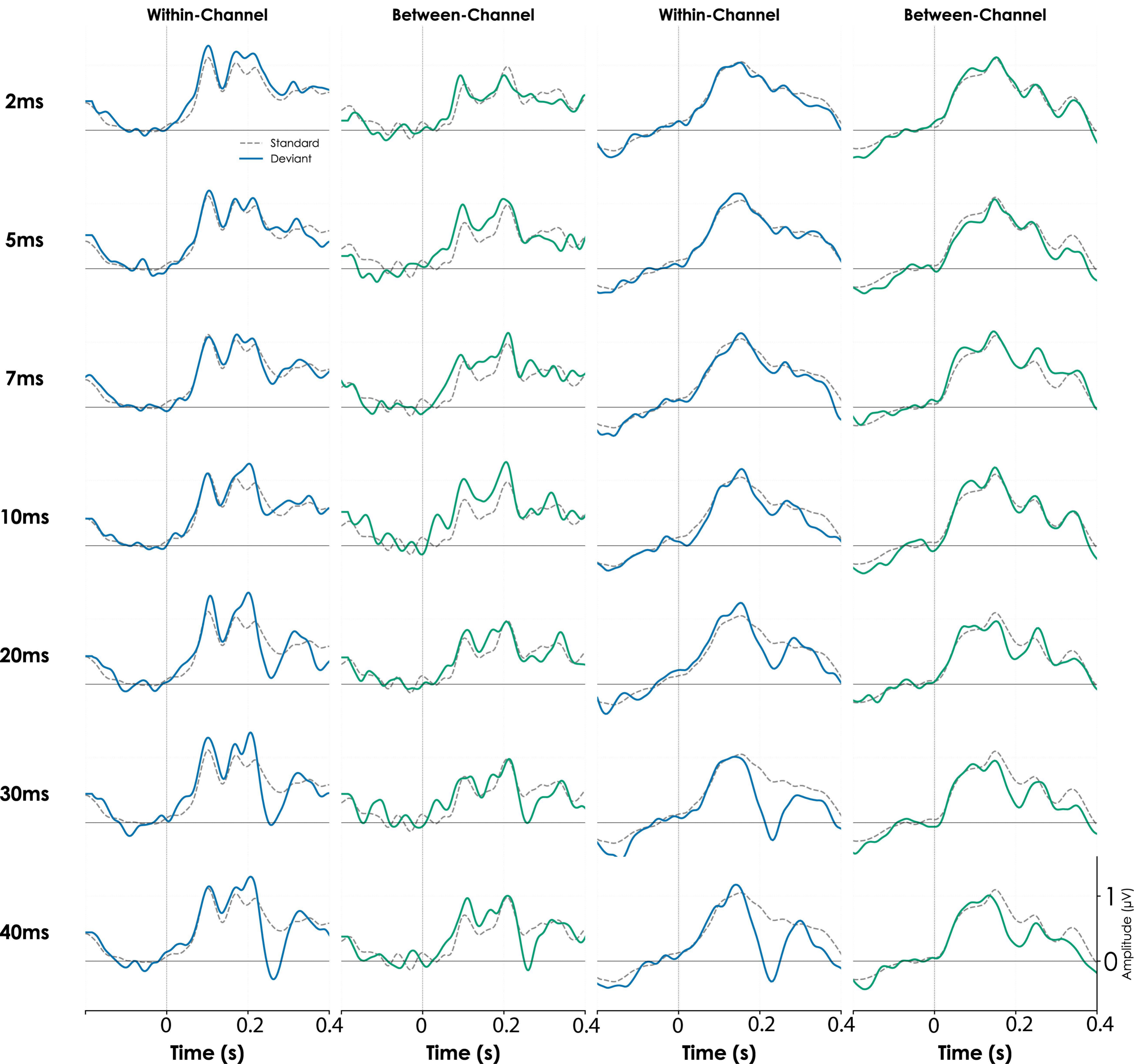

Supplement: Supplementary file 1 — Data S1: Annex.pdf. Annex.: Grand‐average ERP waveforms of younger and older adults across stimulus conditions for various gap durations. Grand‐average ERP waveforms for younger and older adults in within‐channel (blue) and between‐channel (green) gap conditions across seven gap durations (2–40 ms). Each panel shows the deviant (solid line) and standard (dashed line) responses at the fronto‐central region of interest (ROI), comprising electrodes Fz, Cz, FC1, and FC2. Amplitude is plotted in microvolts (μV) and time in seconds (s) with positivity plotted upwards. [file EJN-63-0-s001.pdf]
